# Quality of life after out-of-hospital cardiac arrest: The parisian registry

**DOI:** 10.1186/2197-425X-3-S1-A847

**Published:** 2015-10-01

**Authors:** G Geri, F Dumas, F Bonnetain, W Bougouin, B Champigneulle, M Arnaout, J-D Chiche, F Pène, J-P Mira, J-P Empana, A Cariou

**Affiliations:** Cochin Hospital, Medical Intensive Care Unit, Paris, France; Paris Descartes University, Paris, France; Sudden Death Expertise Center, INSERM U 970, Paris, France; Emergency Department, Cochin Hospital, Paris, France; Besançon University Hospital, Methodological and Quality of Life unit in Oncology, Besançon, France

## Introduction

Numerous studies examined the short-term outcome of resuscitated out-of-hospital cardiac arrest (OHCA) patients, but data on long-term outcome remain scarce. Moreover health-related quality of life (HRQOL) is poorly known in such patients. Our aims were to compare their long-term HRQOL of OHCA with that of the general population, and to assess factors associated with better HRQOL.

## Patients and Methods

We used a large cohort of OHCA patients admitted in a tertiary medical intensive care unit. The main outcome was HRQOL assessed by SF-36 questionnaire. As a comparison group, each OHCA case was matched with 4 controls of same age and sex issued from the decennial survey on population health and medical services involving 25,000 families in France. The associations between pre and intra hospital variables (according to Utstein style) and early interventions (i.e. therapeutic hypothermia and immediate percutaneous coronary intervention) with the different dimensions of the SF-36 questionnaire were investigated using MANCOVA.

## Results

During the study period, 1829 OHCA patients were admitted in our unit. Among them, 602 have been discharged alive. SF-36 interview took place after a median duration of 50 [22-93] months after cardiac arrest. Between discharge and the SF-36 interview, 137 patients died and 211 were lost of follow-up. Thus we collected SF-36 data for 254 patients. Median age was 55 [45,64] years and patients were mostly male (73.6%). Median time from collapse to ROSC was 14 [10,22] min and initial rhythm was shockable in 209 (82.3%) cases. Physical dimensions of SF-36 were more affected than mental dimensions in OHCA survivors compared to French general population and of matched controls from general population (black circle). Physical functioning (74.2 vs. 85.3, p < 0.01), general health (61.1 vs. 67.8, p < 0.01), vitality (50.7 vs. 57.4, p < 0.01), physical-role (71.0 vs. 82.2, p < 0.01) and emotional-role (73.1 vs. 82.0, p < 0.01) were the most altered dimensions. In multivariate analysis, younger age and male gender were associated with almost all SF-36 dimensions. Initial shockable rhythm was associated with a gain in most of the SF-36 dimensions as well. Successful PCI was associated with a gain in physical functioning (+6.71, p = 0.07) and in general health (+6.55, p = 0.04).

(Figure: SF-36 dimensions of 254 OHCA patients (grey rectangles)

## Conclusions

Health-related quality of life is altered in OHCA survivors compared to French general population, especially in physical components of SF36 scale. Younger age, male gender, initial shockable rhythm and coronary reperfusion were independently associated with a better HRQOL.

**Figure 1:**
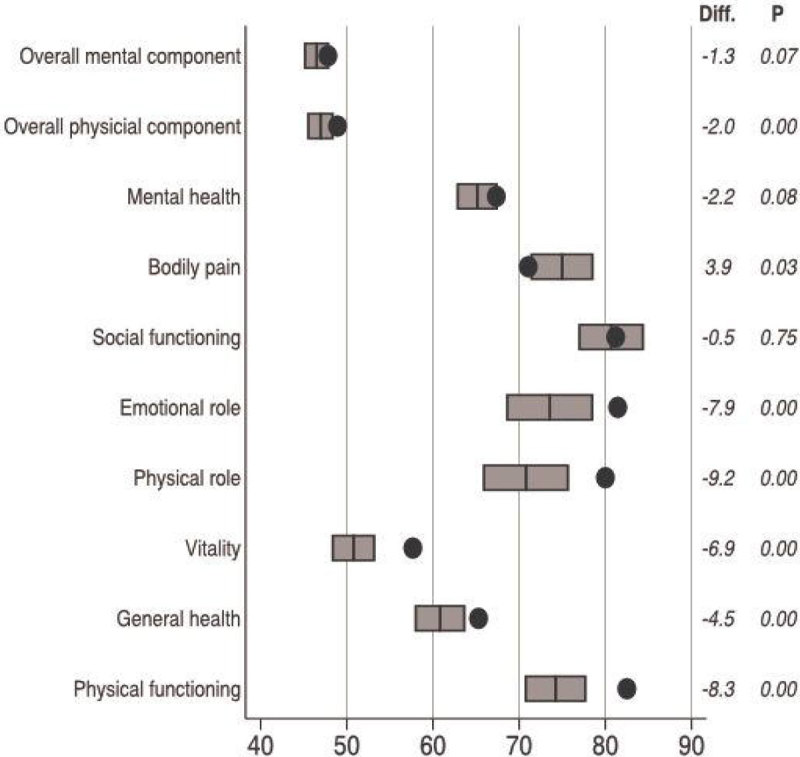
Figure 1

